# Eye-Hand Coordination during Visuomotor Adaptation with Different Rotation Angles: Effects of Terminal Visual Feedback

**DOI:** 10.1371/journal.pone.0164602

**Published:** 2016-11-03

**Authors:** Miya K. Rand, Sebastian Rentsch

**Affiliations:** Leibniz Research Centre for Working Environment and Human Factors (IfADo), Dortmund, Germany; VU University Amsterdam, NETHERLANDS

## Abstract

This study examined adaptive changes of eye-hand coordination during a visuomotor rotation task under the use of terminal visual feedback. Young adults made reaching movements to targets on a digitizer while looking at targets on a monitor where the rotated feedback (a cursor) of hand movements appeared after each movement. Three rotation angles (30°, 75° and 150°) were examined in three groups in order to vary the task difficulty. The results showed that the 30° group gradually reduced direction errors of reaching with practice and adapted well to the visuomotor rotation. The 75° group made large direction errors of reaching, and the 150° group applied a 180° reversal shift from early practice. The 75°and 150° groups, however, overcompensated the respective rotations at the end of practice. Despite these group differences in adaptive changes of reaching, all groups gradually adapted gaze directions prior to reaching from the target area to the areas related to the final positions of reaching during the course of practice. The adaptive changes of both hand and eye movements in all groups mainly reflected adjustments of movement directions based on explicit knowledge of the applied rotation acquired through practice. Only the 30° group showed small implicit adaptation in both effectors. The results suggest that by adapting gaze directions from the target to the final position of reaching based on explicit knowledge of the visuomotor rotation, the oculomotor system supports the limb-motor system to make precise preplanned adjustments of reaching directions during learning of visuomotor rotation under terminal visual feedback.

## Introduction

Various gaze behaviors during manual actions are established through extensive practice in daily life to enable an effective flow of visual information for planning and execution of skillful actions. When reaching movements are made to a target, initiation of saccades to the target usually precedes that of hand movements [1–4]. This gaze behavior is useful to improve reaching accuracy by providing the target information through foveal vision to update the planning of reaching movement [1,2,4–7]. The gaze then remains fixated to the target until reaching is completed [8–12]. This gaze behavior (called gaze anchoring [10]) is useful to control reaching movement, especially during the homing-in phase, because it places the visual target in fovea as the hand approaches to the target, while temporally removing the added burden of spatial updating for gaze shift [13]. Functionally, it allows for effective use of visual feedback of both the target and the approaching hand to guide and complete a precise reaching movement [11,12,14,15].

We have recently examined adaptive changes of gaze behavior during learning of a visuomotor rotation where reaching was made on a digitizer while continuous on-line visual feedback (cursor) of reaching was rotated and displayed on a monitor [16]. In the early learning phase, gazes initially went to the cursor for a substantial part of reaching, and then went to the target. As practice continued, however, the timing of gaze shift to the target was gradually changed to an earlier phase of reaching, and gaze anchoring to the target emerged (see also [17] for complex visuomotor learning). Such an adaptive gaze pattern was similar across different rotation angles used for the visuomotor learning, even though adaptive strategies of hand movements were different depending on those angles, such as implicit adjustments, on-line feedback-based control, and explicit strategic adjustments for the 30°, 75° and 150° rotations, respectively [16]. These observations revealed the robustness and generality of establishing the gaze anchoring to the target during learning of a visuomotor rotation under continuous on-line visual feedback.

It is well known that adaptive changes of reaching also occur in learning of a visuomotor rotation under the terminal visual feedback (or end-point feedback) [18–24]. When continuous visual feedback is available throughout reaching, it allows the sensorimotor systems to compare hand movements and their outcomes (i.e., visual feedback) on-line and to apply visual feedback-based corrections of reaching. Conversely, when terminal feedback is shown after the end of reaching, any corrections of reaching have to be made off-line at the preplanning stage of subsequent trials. Previous studies showed that these two types of visual feedback differentially affected reaching during leaning of visuomotor rotations. Under continuous feedback, reaction time to initiate reaching was shorter [18,19,24], movement time was longer [18,19,22], and precision of target acquisition was higher [22] compared to the learning under terminal feedback. Direction error of a preplanned portion of reaching was also either similar to [18,19] or smaller than [22] that observed under terminal feedback.

Both types of visual feedback used for a visuomotor rotation paradigm can lead to both implicit adaptation and explicit learning [24]. Implicit adaptation is thought to be accomplished by a realignment of a visuomotor map of the relationship between vision and proprioception related to reaching based on implicit knowledge of the visuomotor rotation acquired through practice. Conversely, explicit learning is accomplished by strategic preplanned adjustments of reaching direction through explicit knowledge of the rotation. It is still in debate, however, which type of visual feedback leads to more implicit adaptation of visuomotor transformations. Several studies with visuomotor rotations showed that only continuous feedback led to implicit adaptation [18,19,22,25], whereas another study showed that both types of feedback led to such adaptation but the continuous feedback resulted in greater implicit adaptation than the terminal feedback [24]. Thus, for relatively simple linear visuomotor transformation such as a visuomotor rotation, continuous feedback seems to facilitate more implicit adaptation. On the other hand, for more complex nonlinear visuomotor transformations, terminal feedback seems to facilitate implicit adaptation more than continuous feedback does [26–29].

Despite the above understanding of the effects of visual-feedback types on learning visuomotor transformations, it is unknown whether adaptive gaze patterns during the learning under terminal visual feedback differ from the one under continuous on-line feedback, and our previous study [16] only examined the case of continuous feedback. There is a possibility that the pattern is different at least for the following two reasons.

The first reason is related to the presence or absence of a feedback cursor during reaching, and thus the presence or absence of visual closed-loop control of hand movements. Under concurrent visual feedback, gaze anchoring to the visual target would be beneficial especially for the homing-in phase, where closed-loop control is dominant to precisely guide the feedback cursor toward the visual target (thereby guiding the hand to the task goal) [8,10,12,16,17]. Conversely, this would not be the case under terminal visual feedback. Once reaching is initiated, the feedback cursor disappears, and hence the visual target has no longer any functional role in guiding the cursor toward it. Thus, gaze can be removed from the visual target during reaching under terminal feedback.

The second reason for the potential difference in adaptive gaze pattern under terminal feedback is related to different types of learning involved, i.e., explicit and implicit learning. Previous studies of learning of visuomotor rotations suggest that continuous feedback seems to lead to stronger implicit adaptation of reaching than does terminal feedback [18,19,22,24]. When terminal feedback is applied, adjustments of reaching have to be made off-line at the preplanning stage of subsequent trials, and thus, the gaze pattern might be adapted to looking at a location that would benefit explicit preplanning of reaching under a visuomotor rotation. Such a location would likely be the final position of reaching movement that counters the applied rotation. This is so because our previous study showed that gazes naturally went to that direction when participants were informed of the nature of a visuomotor rotation from the start and instructed to apply an explicit strategy to counter it [21]. Therefore, when explicit adjustments of reach direction are gradually established through learning, the gaze pattern should also be gradually adjusted toward that direction. Such an adaptive gaze pattern would mean that spatial alignment of gaze on the final position of reaching is functionally important for preplanning, and hence the oculomotor system tries to restore it during learning of a visuomotor rotation.

The present study investigated adaptive changes of gaze behaviors by using terminal feedback during learning of a visuomotor rotation. One hypothesis was that the gaze anchoring to the visual target is maintained throughout practice. This would imply that control of already established gaze anchoring behavior to a visual target is independent of adaptive processes related to a visuomotor rotation under terminal feedback. An alternative hypothesis was that gazes are directed to the visual target in the early practice phase, but as practice progresses, they gradually shift in the direction of the final position of reaching that counters the applied rotation. This would imply that the oculomotor system re-establishes gaze anchoring in relation to the final position of reaching instead of to the visual target in order to facilitate planning of hand movements under a visuomotor rotation.

Similar to our previous study that used continuous feedback [16], the present experiment utilized three rotation angles (30°, 75°, and 150°) to vary the difficulty of the visuomotor transformation. Based on previous studies [16,30–33], adaptation to the 30° rotation is expected to be easier than that to the 75° rotation simply because of the smaller rotation angle; both rotations would involve one rotational transformation. In contrast, the 150° rotation would involve a two-step transformation [16,30–32]. The first step is a relatively easy 180° polarity inversion of both axes (i.e., a reversal shift [32]), and the second step is a “backward” shift to the rotated visuomotor map. Accordingly, the involvement of the reversal shift makes the difference between the 150° and 30° rotations, but the former with a reversal shift would be more difficult than the latter. Therefore, utilization of the three rotation angles provided us an opportunity to examine if gaze patterns during adaptations of visuomotor rotations with terminal feedback may differ depending on the difficulty of the transformations.

## Materials and Methods

### Participants

Thirty six healthy, young, right-handed adults (mean ± SD: 23.5 ± 3.2 years old, 18 females and 18 males) signed written informed consent and participated in the study. All participants self-reported being free of neurological or sensory impairments. They had normal color vision according to the Ishihara test [34] and normal visual acuity. The participants were randomly assigned to one of three groups (30°, 75° or 150° of visuomotor rotation, 12 participants each). The present study was approved by the ethics committee of the Leibniz Research Centre of Working Environment and Human Factors. The study was conducted in accordance with the ethical standards laid down in the 1964 Declaration of Helsinki.

### Apparatus

The experimental setup was similar to that of our previous study [16]. Participants were comfortably seated on a height-adjustable chair in front of a table, on which a digitizer (Wacom Intuos 4XL, active area size: 488 x 305 mm) and a vertical 22-inch computer monitor (Dell P2210, flash rate: 60 Hz) were placed. An infrared eye-tracking system (iViewX 500 RED, SMI) was attached underneath the monitor. The participants rested their chin on a chin rest. The distance between participants’ eye position and the center of the monitor was 628 ± 21 (SD) mm on average. A starting position (SP, black circle, 6 mm in diameter) displayed in the center of the monitor was aligned with the participants’ median plane. Participants held a stylus with their right hand in a manner like holding a pen for handwriting, and moved their hand in a horizontal plane on the digitizer. An opaque board placed 170 mm above the digitizer surface blocked participants’ view of their hand movements. Terminal visual feedback of each hand movement was displayed as a cursor (a red circle, 4 mm in diameter) on the monitor after the movement. Hand movements of the stylus on the digitizer and those of the cursor on the monitor had a one-to-one ratio with respect to distance.

For hand movements, the x- and y-positions of the tip of the stylus were recorded by the digitizer at 200 Hz with a spatial resolution of 0.005 mm. The x- and y-positions of eye movements were recorded at 500 Hz by the eye tracker. The eye tracker recorded the left and right gaze positions separately and returned the arithmetic mean of the two gaze positions as data output. For each of the x- and y-positions, spatial resolution of the eye tracking system was 0.03° and its accuracy was 0.4°. The eye tracker was calibrated for each participant before data recording by using nine calibration points displayed across the monitor. For synchronization of eye and hand data recording, data acquisition software written in C++ simultaneously initiated the recording of the digitizer and eye tracker.

### Procedure

**Fig 1:**
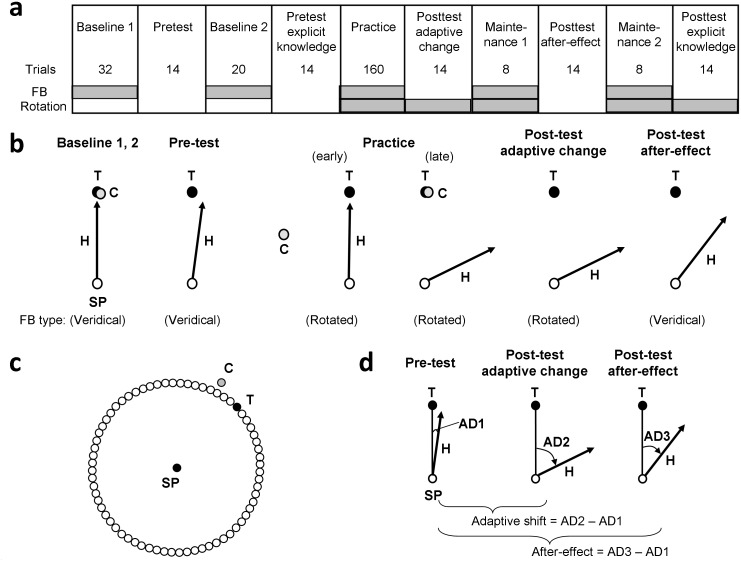
Experimental conditions and schematic illustrations of some conditions and a target display. In (a), the order of all experimental conditions and respective numbers of trials used are shown. Conditions where visual feedback (FB) of hand movement was present and/or visual feedback was rotated (Rotation) are marked in shaded squares. Participants were informed of the type of visual feedback at the beginning of each condition. For the conditions where the display of visual feedback was absent, participants were instructed to perform the task as if it were in a veridical-feedback environment or in a rotated-feedback environment. In (b), there are illustrations of selected experimental conditions, where hand movement (H), the starting position (SP), the target (T), and the feedback cursor (C) are shown. In (c), there is an illustration of the SP, target, and cursor display. In (d), there is an illustration of calculating adaptive shift and after-effect based on the pre-test, post-test adaptive-change and post-test after-effect for hand movements. The angular deviation (AD) is calculated as the direction of hand movement from the line connecting the hand position at movement onset and the center of the target. Adaptive shift (or after-effect) is calculated by subtracting AD1 from AD2 (or AD3).

On an invisible circle with a radius of 100 mm, 104 black open circles (6 mm in diameter) were placed (Fig 1c), forming a large ring around the SP. This ring was introduced so that gaze was not externally attracted by a single visual target in the working space. Four targets (black circles, 6 mm in diameter) were displayed on four of the above open circles in the directions of 45°, 135°, 225°, and 315°. The resulting visual angle between the SP and target positions was 9.05°. Note that the 3 o’clock position was 0° and the counterclockwise (CCW) direction had a positive sign.

Similar to previous studies [16,21,35–38], at the beginning of each trial, participants were guided to the SP by one or two out of four red arrows pointing to the left, right, up, and down, appearing in the margins of the monitor. The idea behind this procedure was to guide participants to the SP without providing visual feedback of specific hand positions. The SP and the feedback cursor became visible when the stylus was within a radius of 15 mm from the SP, so that the participants were able to reach the SP reliably. Note that this visual feedback was veridical. After the cursor had stayed at SP for 500 ms, the black open circles arranged on the invisible ring appeared (Fig 1c). After a random interval between 500 to 1300 ms, one of the four open circles in the target directions was changed to a filled black circle (Fig 1c, T), which served as a target for reaching, and the SP and the feedback cursor disappeared.

In response, the participants made a reaching movement to the target. Participants were instructed to move as quickly and as accurately as possible to reach the target. No feedback cursor was shown during the movement. The completion of the movement was automatically detected by computer software when the distance between successively sampled positions of the stylus remained equal to or less than 0.25 mm for 400 ms. After the detection of hand-movement completion, the feedback cursor reappeared and stayed at the same location for 1500 ms, serving as a terminal visual feedback. After the feedback cursor had been displayed, the trial ended.

### Experimental conditions

Similar to previous studies [16,35,36], we used ten fixed-order conditions (Fig 1a): (1) baseline 1, (2) pre-test, (3) baseline 2, (4) pre-test explicit-knowledge, (5) practice, (6) post-test adaptive-change, (7) maintenance 1, (8) post-test after-effect, (9) maintenance 2, and (10) post-test explicit-knowledge. The number of trials recorded for each condition is shown in Fig 1a. Terminal visual feedback was displayed on the monitor after the completion of hand movement only for conditions of baseline 1 and 2, practice, and maintenance 1 and 2 (Fig 1a, FB). For conditions where a visuomotor rotation was applied (i.e., practice, post-test adaptive-change, maintenance 1 and 2, and post-test explicit-knowledge, see Fig 1a, rotation), visual feedback was rotated 30°, 75° or 150° in the CCW direction depending on experimental groups. Veridical visual feedback was used for the rest of conditions (Fig 1a, rotation). The participants were informed of the type of visual feedback by the examiner at the beginning of each condition. For all conditions with veridical feedback, participants were informed that the reaching task was easy, but no information about the nature of visual feedback (i.e., veridical) was provided. For all conditions with rotated visual feedback, participants were informed that the reaching task was more difficult than the easy one, but no information about the introduction of a visuomotor rotation was provided. For the conditions where the display of visual feedback was absent, participants were instructed to perform the task as if it were an easy one (i.e., veridical visual feedback for pre-test and pre-test explicit-knowledge, and for post-test after-effect) or a more difficult one (i.e., rotated visual feedback for post-test adaptive-change and post-test explicit-knowledge).

In the baseline 1 condition (Fig 1b), the participants made rapid reaching movements from the SP to the target; and after the movement, a feedback cursor indicated the final position. The participants were instructed that the goal of their task was to reduce the distance between the red feedback cursor and the black target so that the cursor became aligned with the target. After the baseline 1 condition, the pre-test (Fig 1b) was performed under the same procedure except that no visual feedback was provided at the end of each trial. Pre-test was used to examine baseline performance under no terminal visual feedback of the hand movement. Next, the baseline 2, which was same as baseline 1, was performed. Afterwards, the participants performed the pre-test explicit-knowledge, which examined the baseline level of participants’ explicit knowledge of reaching direction to the target. For this purpose, we used a procedure similar to the ones used in previous studies [16,21,35,38,39]. The SP and the target were presented together with a line starting from the SP. The line was slightly shorter than the SP-target distance. The line was initially presented at an angle of 150° clockwise (CW) from the SP-target direction, and it slowly moved around the SP. The participants instructed the experimenter to stop the movement of the line at the orientation that they judged to be correct as the reaching direction to the target under the absence of visuomotor rotation. As needed, the orientation of the line was further adjusted (backward and/or forward) by participants verbally instructing the experimenter to start and subsequent stop the movement of the line until they were satisfied with the orientation. The pre-test explicit-knowledge was followed by the practice condition with a visuomotor rotation (Fig 1b).

After the practice condition, three types of post-tests (adaptive-change, after-effect, and explicit knowledge, Fig 1a) were carried out to assess different types of knowledge of the visuomotor rotation acquired through practice [16,26,39,40]. Post-test adaptive-change (Fig 1b) was performed under the presence of visuomotor rotation, but no visual feedback was provided. This test was identical to the pre-test except for the presence of visuomotor rotation, of which the participants were informed. The changes of movement direction from the pre-test to the post-test adaptive-change were measured as adaptive shifts (Fig 1d), which reflected both implicit adjustments and explicit strategic adjustments of hand directions. Post-test after-effect (Fig 1b) was identical to the pre-test and differed from the post-test adaptive-change with respect to the absence of rotation. The participants did not see the terminal feedback of their hand movements on the monitor, but were informed that the visuomotor rotation was no longer present. Hence, there was no reason for the participants to apply explicit strategic adjustments to compensate for the rotation. Thus, any changes of movement direction from the pre-test to the post-test after-effect condition were measured as after-effects (Fig 1d), which reflected only implicit adjustments of hand directions acquired through practice.

Post-test explicit-knowledge was identical to the pre-test explicit-knowledge except that the participants judged the direction of the line that matched the reaching direction for bringing the cursor to the target under the presence of visuomotor rotation. Since the participants knew about the presence of a visuomotor rotation in the post-test explicit-knowledge condition, any changes of judgments of movement direction from the pre-test explicit-knowledge condition (which was performed under the absence of the rotation) reflected explicit knowledge of the rotation acquired through practice. These changes were measured as explicit shifts. Between the post-tests, participants performed maintenance 1 and 2 conditions, respectively, which were identical to the practice condition.

### Data analysis

#### Analysis of hand movements

The x- and y-positions of the stylus were upsampled at 500 Hz with a custom program written in Matlab (Mathworks); the raw data was first interpolated with a method of Piecewise cubic Hermite interpolation [41] and then resampled at 500 Hz. Subsequently, these data were filtered with a Butterworth filter (low-pass, 4^th^ order, 10 Hz cutoff frequency), and differentiated by using a two-point central-difference algorithm to obtain velocity. Calculations of onset and offset of hand movements were performed by an automated movement parsing algorithm ([42] algorithm B). The results of this automatic procedure were inspected and corrected manually as needed based on visual inspection; 9.4% (onset) and 15.7% (offset) of all analyzed trials were corrected.

Reaction time was measured from the target onset to the onset of hand movement. Movement time was measured from the onset to the offset of hand movement. Direction error of the hand movement was measured in the baseline 2 and practice conditions. First, a reference line was defined as the line connecting the hand position at movement onset and the center of the target (in the case of baseline 2) or the line connecting that onset position and the hand position that would place the cursor on the target center under the rotated feedback (in the case of practice). The direction error was computed as the angular deviation from the reference line to the line connecting the hand positions at movement onset and offset. The direction error was positive (or negative) when a hand-path was directed CCW (or CW) to the reference line.

Adaptive shift, after-effect, and explicit shift were measured by comparing results of pre-tests and post-tests. Angular deviation of the line connecting the hand positions at movement onset and offset from the line connecting the hand onset position and the target center was computed for each trial of the pretest, post-test adaptive-change and post-test after-effect (Fig 1d). Subsequently, the difference between circular mean [43] of angular deviation across all trials of post-test adaptive-change (or post-test after-effect) and that of pre-test was calculated as adaptive shift (or after-effect) for each participant. For each trial of pre- and post-tests of explicit-knowledge, a judged rotation angle was computed as the angular deviation of the direction of the line, as judged by the participants, from the direction of the SP-target line. Subsequently, explicit shift was measured as the difference between circular mean [43] of judged rotation angle across all trials of the pre-test explicit knowledge and that of the associated post-test in each participant.

#### Analysis of eye movements

Analyses of eye movements were carried out based on unfiltered x- and y- position data to avoid any types of distortion of signal through filtering because of saccadic eye movements being very fast. Eye movements were differentiated by using a two-point central-difference algorithm to obtain velocity. Onset and offset of the initial saccade immediately after the target presentation were determined using a threshold criterion of 20 cm/s in the velocity profile [8,11,12]. The results of this automatic procedure were inspected and corrected manually as needed based on visual inspection; 27.7% (onset) and 22.8% (offset) of all analyzed trials were corrected.

All parameters of eye movements related to direction errors, adaptive shifts and after-effects were analyzed in similar manners as described above for hand movements. The only difference was that while the hand-related parameters were measured based on the line connecting the onset and offset of hand movements, the eye-related parameters were measured based on the line connecting the center of SP and the gaze position at the onset of hand movement. Note that at this timing, gazes were already shifted away from the SP, and gaze shifts associated with the execution of hand movements were also completed in most of the trials.

**Fig 2:**
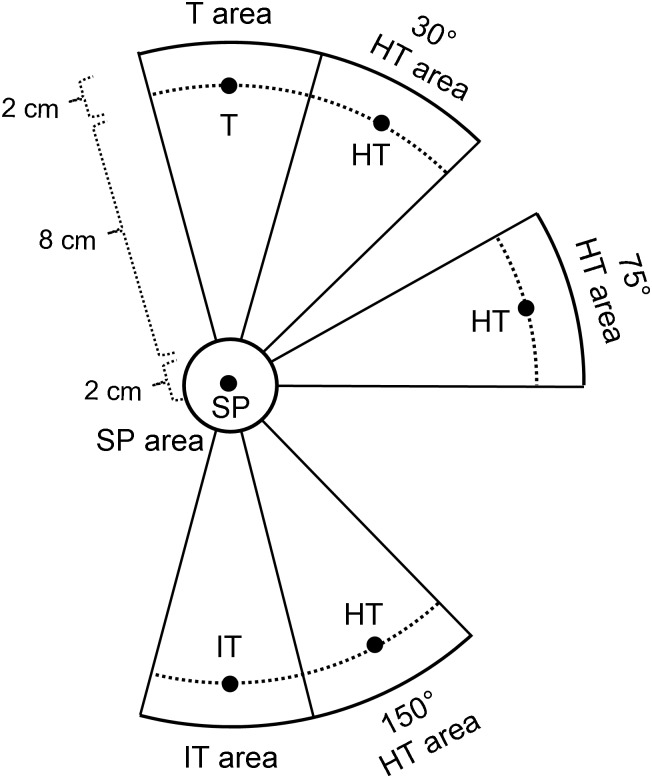
Different gaze areas used for analysis of gaze locations. Six black dots indicate the starting position (SP), the target (T), the hand targets (HT, one for each feedback-rotation group), and the inverted target (IT) positions. The HT position is the hand location that would bring the cursor to the target under the rotated visual feedback. The IT position is the 180° inverted location of the target. There are five gaze areas (T area, HT area, IT area, SP area, and other area that is the remaining area of workspace) for each group.

Adaptive change of gaze locations associated with the execution of hand movements was further examined in terms of gaze incidences at various areas of the workspace, similar to our previous study [21]. We defined 5 gaze areas for this analysis: the target (T) area, the hand-target (HT) area, the inverted-target (IT) area, the SP area, and the other area (Fig 2). The SP area was a circular field around SP (2 cm in radius). Different sections of another, larger circular area centered on the SP (12 cm in radius) and excluding the SP area served as the T, HT and IT areas, each of which ranged between ± 15° with the central direction defined by SP-T, SP-HT, and SP-IT lines, respectively. The HT area was related to the hand position (HT, Fig 2) that would bring the cursor to the target center under the rotated visual feedback. Thus, each group had its own HT area. The inverted-target area was related to the position (IT, Fig 2) where the target was inverted by 180°. The remaining workspace was defined as the other area. We identified to which area gazes went among these 5 gaze areas in each trial. This identification was performed at each of the following 6 timings associated with the execution of hand movements in each trial: 200 ms and 100 ms prior to the movement onset, at the movement onset, 100 ms after the movement onset, peak velocity, and at the movement offset. Subsequently, the number of incidents was counted for each gaze area and the incidence was calculated as the percentage of trials for baseline performance and for each block of trials for practice. Note that these measurements included both gaze fixation and saccade (if a saccade happened to occur at the time of measurement). However, gaze was typically fixated by 100 ms prior to the movement onset and maintained until the movement offset; and thus, the measurements at the time of the movement onset reported in the Results section were based on gaze fixation in most of the trials.

In addition to the above analysis of gaze locations associated with the execution of hand movement, we also examined gaze patterns immediately after the target presentation. The initial saccade that occurred in the period from the target presentation to the onset of hand movement was analyzed in each trial of the practice condition. We identified to which area gaze went with the initial saccade. This was determined based on the gaze fixation location measured at the offset of the initial saccade. Subsequently, the gaze incidence for each gaze area was calculated as the percentage of all trials of practice that contained such initial saccades.

Trials with bad hand recording (e.g., missing data points) or bad eye recording (e.g., blinking, artifact) that occurred at the onset of hand movement or prevented the identification of the initial saccades were eliminated from the further analysis (388 trials: 4.85% of all trials across conditions of baseline 2, practice, pre-test, post-test adaptive-change, and post-test after-effect). For analysis of pre-test, and post-test adaptive-change and post-test after-effect, the data of hand movements was further screened for outliers for each test and each group by calculating mean and standard deviation (SD) across all trials of all participants. Twelve trials (0.83%) outside the range of mean ± 3SD were identified as outliers and further eliminated from the analysis. The 3SD was chosen empirically by observing each of all histograms of pre- and post-tests, so that only outliers that were clearly separated from the main distribution were eliminated. For analysis of pre- and post-tests explicit knowledge, data was also screened for outliers in the same way as explained above, and 20 trials (3.97%) were eliminated from the analysis.

### Statistical analysis

For examination of baseline performance, the last 8 trials of the baseline 2 condition were used for all parameters except for the trial-based correlation between eye and hand movements, for which the last 16 trials of the baseline 2 condition were used. For the practice condition, all trials were mainly divided into 40 blocks consisting of 4 trials each to keep a reasonably high resolution for observing adaptive changes. Exclusively for analysis of trial-based correlation between eye and hand movements, all practice trials were divided into 10 blocks consisting of 16 trials each. The 16 trials/block was chosen to secure enough sample sizes for calculating the correlation within each block and participant even if some trials where gaze fell in the SP (Fig 2) area were removed for this analysis. Furthermore, the first 2 blocks (8 trials) and the last 2 blocks (8 trials) of the practice condition were defined as early and late phases of practice, respectively, for all related analyses except the correlation analysis, for which the first block (16 trials) and the last block (16 trials) were defined as the early and late phases, respectively.

For baseline performance as well as each trial block and each phase of the practice condition, the following parameters were calculated in each participant: (1) Mean reaction time and mean movement time were calculated across trials. (2) Incidence of gazes at each gaze area was calculated as the percentage of trials. (3) Circular mean and circular variance [43] were calculated across trials for hand direction error and gaze direction error. Note that circular variance had an arbitrary unit with 1 being the maximum and 0 the minimum. (4) To examine the relation between gaze and hand directions, circular-linear correlation [43] between gaze direction error and hand direction error was calculated among trials.

Subsequently, these individual values were used to calculate each group’s mean in baseline performance as well as in each trial block and each phase of the practice condition. Exclusively for individual circular means of direction errors of hand movement, the group’s mean and 95% confidence interval of the mean were calculated using circular statistics [43], the reason being that individual circular means of some groups still had large ranges of distribution (> 180°) and included a discontinuity at +/- 180°.

For all of the above parameters, except gaze incidence at each gaze area and the circular mean of hand direction error, the baseline performance and adaptive changes through practice were assessed by using a 3 (between-subject factor group: 30°, 75° and 150°) x 3 (within-subject factor phase: baseline, early practice, and late practice) ANOVA.

For the parameter of circular mean of hand direction error, the 3 x 3 ANOVA, however, could not be used because the distribution of individual circular means of the early phase of some group(s) had a large range (> 180°) and included the discontinuity at +/- 180°. For this reason, circular statistics were used for testing group difference of the early phase. Furthermore, to examine a possible group-by-phase interaction during practice, a difference score was calculated between the early and late practice phases in each participant, and this value was also subjected to circular statistics for testing group difference. Since data of the 75° group did not deviate from circular uniformity (Rayleigh test: *p* > 0.05 [43]), these group differences were assessed by using a multi-sample test for equal median directions [43]. Moreover, the group difference of hand direction error was assessed separately in the baseline and late practice phase by using a one-way ANOVA. To assess whether movements at the end of the practice condition differed from baseline performance, the mean value of baseline performance and that of the late practice phase were compared by using a paired t-test in each group.

For the parameter of gaze incidence, analyses were focused on adaptive changes during practice for succinct presentations of results for all 5 gaze areas. A 3 (between-subject factor group: 30°, 75° and 150°) x 2 (within-subject factor phase: early practice, and late practice) ANOVA was used to test group and phase differences. This ANOVA was performed on the arcsine-transformed incidence values to address the non-normality of proportions [44]. Furthermore, the progressive changes in gaze incidences during practice were quantified using a linear regression analysis in each group and each of T, HT, IT and other gaze areas. For the regression, group’s mean incidence was used as dependent variable and the block of trials was used as an independent variable. In addition, a one-way ANOVA with between-subject factor group was used to assess group differences in gaze incidences at the target area during baseline performance as well as those related to initial saccade observed during practice.

When appropriate, post-hoc comparisons were performed using Bonferroni corrected t-tests (or a two-sample test for equal median directions [43]) in terms of a main group effect or phase effect found in the ANOVAs (or in the multi-sample test for equal median directions). When a group-by-phase interaction was significant from the 3 (group) x 3 (phase) ANOVA, post-hoc comparisons were performed using Bonferroni corrected 3 (group) x 2 (phase) ANOVAs to identify which pair among the 3 phases included a significant group-by-phase interaction. Mauchly’s test was used to determine whether sphericity could be assumed. Greenhouse-Geisser epsilon was reported for the results of ANOVA only when Mauchly’s test was found to be significant.

Regarding adaptive shifts, after-effects, and explicit shifts, circular means [43] were calculated across participants in each group. Results of these parameters were examined in each group by using t-tests; specific comparisons are stated in the Results section. Correlations between individual adaptive shifts and individual explicit shifts were also calculated in each group. One exception to those analyses was the data involving the adaptive shift of eye movement for the 150° group, which showed a large range of data distribution across participants, requiring circular statistics. A Watson-Williams two-sample test [43] and circular-linear correlation [43] were used instead of the t-test and correlation, respectively. The probability level for statistical significance was *p* < 0.050. For post-hoc analyses, however, it was set at *p* < 0.016 (i.e., 0.05/3) due to the multiple comparisons.

## Results

We will first report adaptive changes of hand movements due to practice of the visuomotor rotation, followed by those of gaze locations. Subsequently, spatial relation between hand and gaze directions will be reported, followed by results of the pre- and post-tests for hand and eye movements.

### Adaptive change of hand movement

#### Reaction time and movement time

Mean reaction times in baseline performance and the early phase and late phase of practice were 805 ± 76 (SE), 1316 ± 202, and 1208 ± 177 ms for the 30° group, respectively. Comparable values were 652 ± 76, 1318 ± 78, and 864 ± 50 ms for the 75° group, and 621 ± 69, 1108 ± 146, and 1128 ± 194 ms for the 150° group. A 3 (group) x 3 (phase: baseline, early practice, and late practice) ANOVA revealed a significant phase main effect (*F*(2,66) = 16.80, *p <* 0.001, ɛ = 0.715), whereas group (*p* = 0.338) and group-by-phase interaction (*F*(4,66) = 1.07, *p* = 0.378, ɛ = 0.715) effects were not significant. As expected from previous studies [18,19,45], mean reaction times significantly increased from the baseline to the early practice phase (post-hoc, *p* < 0.001). Reaction times in the late practice phase were similar to those in the early practice phase (*p* = 0.156) but greater than those of the baseline (*p* < 0.001).

Mean movement times for the 30° group were 1319 ± 120 (SE), 1199 ± 85, and 1072 ± 127 ms in the baseline and early phase and late phase of practice, respectively. Comparable values were 1167 ± 93, 1141 ± 129, and 981 ± 101 ms for the 75° group, and 872 ± 80, 1031 ± 173, and 911 ± 83 ms for the 150° group. The phase main effect was significant (3 x 3 ANOVA, *F*(2,66) = 3.67, *p* = 0.031, ɛ = 0.803), but there were no group (*p* = 0.190) and group-by-phase interaction effects (*F*(4,66) = 1.48, *p* = 0.217, ɛ = 0.803). Movement times in late practice were similar to those in early practice (post-hoc, *p* = 0.060) but shorter than those in the baseline (*p* = 0.012). The values in the early phase did not differ from those in the baseline (*p* = 0.930).

#### Direction errors

**Fig 3:**
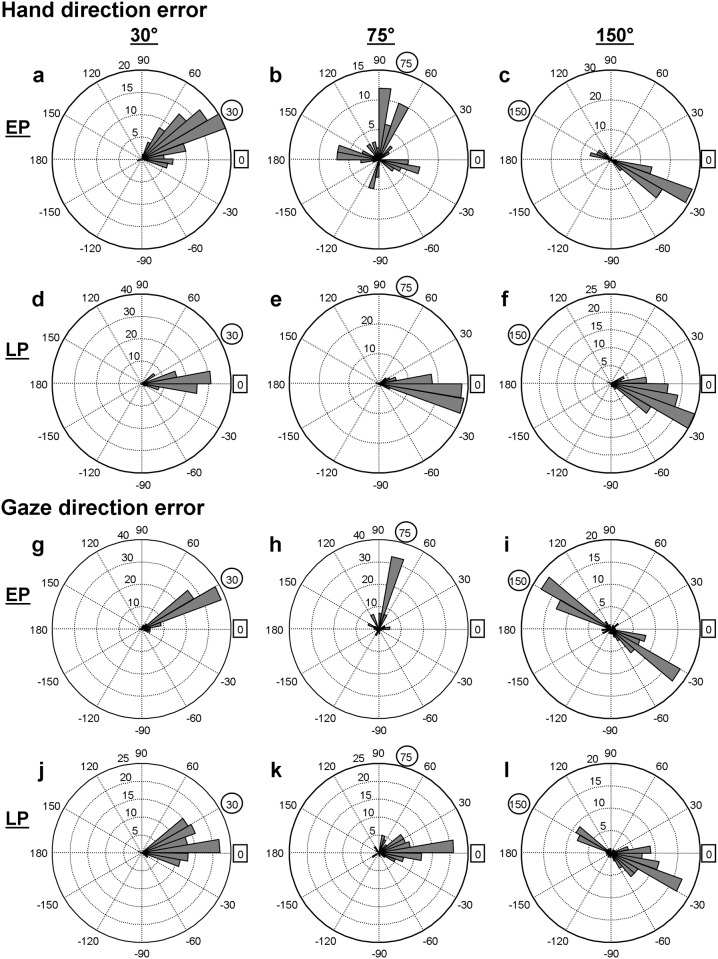
Angular histograms of direction error of hand and eye movements in early and late practice of a visuomotor rotation. Histograms of the 30°, 75°, and 150° groups are shown in the left, center and right columns, respectively. EP refers to the early phase (a-c, g-i), and LP refers to the late phase (d-f, j-l) of practice. The direction labels marked with circles indicate the direction of the target, and the labels with squares indicate direction of the hand target (i.e., the hand location that would bring the cursor to the target under the rotated visual feedback). The binning size of each plot is 10°.

To illustrate adaptive changes of hand movements, angular histograms of the direction errors in the early and late practice phases are shown in Fig 3a–3f. In the early phase, the 30° group (Fig 3a) generally moved their hands in the direction of the target (30°). The 75° group made a wide range of direction errors, which formed roughly four peaks of the distribution with intervals of ~90° from the target direction (75°: Fig 3b), suggesting that the participants explored the reaching directions along and perpendicular to the SP-target line. The 150° group mainly moved their hands to the 180° inverted location of the target (-30°) and less frequently to the target (150°, Fig 3c). In contrast to the early phase, all groups produced one-peak distribution profiles in the late phase. The peak distribution of the 30° group was close to 0° (Fig 3d), whereas respective peak distributions of the other groups were slightly (75°: Fig 3e) or further (150°: Fig 3f) away from 0°.

**Fig 4:**
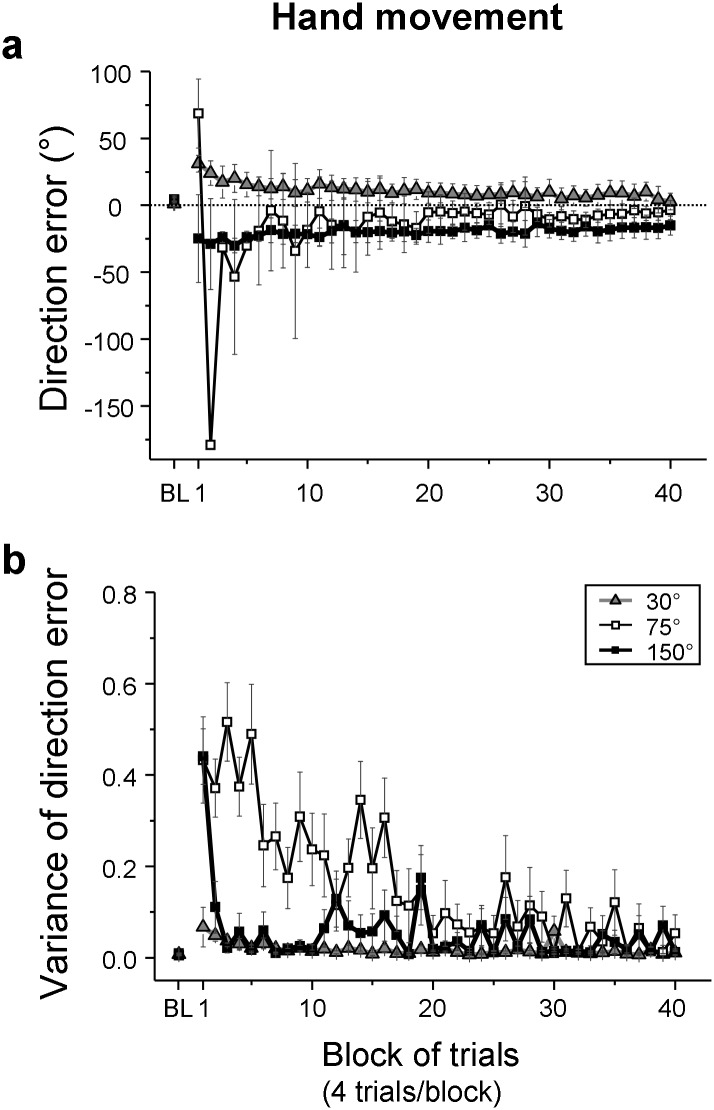
Adaptive changes of hand movements during practice of a visuomotor rotation. Direction errors of reaching (a) and inter-trial variability (i.e., circular variance) of the direction errors (b) are shown. Mean values of all participants are plotted against 40 trial blocks with 4 trials each for the 30° (triangles), 75° (open squares), and 150° (filled squares) groups. Mean values of baseline performance (BL) are also plotted. In (a), the error bars represent a 95% confidence interval. The confidence intervals of the 2^nd^, 3^rd^, and 5^th^ blocks of the 75° group are missing because too large inter-subject variability prevented a reliable calculation. In (b), the error bars represent the SE. Circular variance has an arbitrary unit with 1 being the maximum and 0 the minimum.

Fig 4a shows adaptive changes of mean directions errors throughout practice. In baseline performance without the visuomotor rotation, mean direction errors were small in all groups. Although the 30° group made slightly smaller errors than did the other groups, the differences were not significant (one-way ANOVA, *F*(2,33) = 3.54, *p* = 0.040; post-hoc, 30° vs 75°: *p* = 0.096, 30° vs 150°: *p* = 0.072). All groups substantially increased the direction errors immediately after the introduction of the visuomotor rotation. Thereafter, the 30° group gradually reduced the errors toward 0° throughout practice (Fig 4a, triangles). The 75° group (Fig 4a, open squares) showed very large errors in the first several blocks with large inter-trial (see below, Fig 4b) and inter-subject variability (see error bars of Fig 4a). The mean direction errors were negative for most blocks and gradually adjusted toward 0°. In late practice, the error was still slightly below 0°, indicating some overcompensation of the visuomotor rotation. The 150° group reduced the errors rapidly toward -30° in the early practice phase (Fig 4a filled squares), showing an early implementation of a reversal shift of hand movements to the 180° inverted-target location. The negative direction errors (Fig 4a filled squares) were slightly adjusted toward 0° during practice, but the adaptation was incomplete by the end of practice.

In the early phase, the 30° group had significantly greater direction errors than did the other two groups (multi-sample test for equal median directions: *X²*(2) = 12.66 *p* = 0.002, post-hoc, 30° vs 75°: *p* = 0.014, 30° vs 150°: *p* < 0.001), which did not differ from each other (*p* = 0.102). In the late phase, however, the direction errors of the 30° group were similar to those of the 75° group (one-way ANOVA, (*F*(2,33) = 7.92, *p* = 0.002; post-hoc, *p* = 0.361) but still different from those of the 150° group (*p* = 0.001). Again, the latter groups did not differ from each other (*p* = 0.073). To examine if there was an interaction effect between the group and practice phase, difference scores between the early and late phases were calculated. Mean value for the 150° group (-17.5 ± 28.9° (95% confidence interval)) differed significantly from the other two groups (30° group: 23.6 ± 5.6°; 75° group: 90.4 ± 61.8°; multi-sample test for equal median directions, *X²*(2) = 12.66 *p* = 0.002; post-hoc, 150° vs 30°: *p* < 0.001, 150° vs 75°: *p* < 0.014), which did not differ from each other (*p* = 0.041). The direction errors in the late phase (LP) significantly differed from those of the baseline (BL) for the 75° (LP: -4.4° (circular mean), BL: 4.2°, *t*(11) = 2.61, *p* = 0.024) and 150° groups (LP: -16.0°, BL: 4.4°, *t*(11) = 5.67, *p* < 0.001), but not for the 30° group (LP: 3.5°, BL: 1.1°, *p* = 0.558).

#### Variability of direction errors

Adaptive changes of the inter-trial variability (i.e., circular variance) of hand directions during practice are shown in Fig 4b. Mean variability was small in the baseline performance for all groups (30°: 0.007, 75°: 0.005, 150°: 0.009). Just after the introduction of the visuomotor rotation, the variability increased modestly for the 30° group and dramatically for the 75° and 150° groups. Thence, the 150° group immediately reduced the variability, but the 75° group maintained large variability until the midway of practice. In late practice, however, the variability of all groups was small. A 3 (group) x 3 (phase: baseline, early practice, and late practice) ANOVA showed a significant group-by-phase interaction (*F*(4,66) = 13.99, *p* < 0.001, ɛ = 0.559). The interaction stemmed from differential variability increases among groups from the baseline to early practice (post-hoc, interaction effect of a 2 (phase) x 3 (group) ANOVA, *F*(2,33) = 15.06, *p* < 0.001) and differential decreases among groups from early to late practice (post-hoc, interaction effect, *F*(2,33) = 13.92, *p* < 0.001). As can be seen in Fig 4b, the 75° group made substantial changes from the baseline to early practice and then to late practice, whereas these changes were much smaller for the 150° group and minimum for the 30° group. There were no such differential changes among groups from the baseline to late practice (post-hoc, interaction effect, *F*(2,33) = 0.81, *p* = 0.453). There was also a significant group main effect (*F*(2,33) = 13.65, *p* < 0.001). The 30° group had significantly smaller variability than did the 75° (post-hoc, *p* < 0.001) and 150° (*p* = 0.004) groups, which did not differ from each other (*p* = 0.040). The phase main effect was also significant (*F*(2,66) = 67.39, *p* < 0.001, ɛ = 0.559). The variability in early practice was significantly greater than that of the baseline (post-hoc, *p* < 0.001) and that of late practice (*p* < 0.001). The variability of the baseline and late practice did not differ from each other (post-hoc, *p* = 0.030).

In summary, the 30° group gradually reduced direction errors throughout practice. The 75° group had large direction errors in the first half of practice, and the 150° group applied a 180° reversal shift from early practice; both groups overcompensated the respective rotations. All groups reduced the variability of direction errors to minimal in late practice.

### Adaptive change of eye movement

#### Gaze locations

When the target appeared on the monitor in each trial, the participants usually made a saccade in response. The percentages of trials in the practice condition that contained such initial saccade were 99.4 ± 0.2 (SE) % (30° group), 90.8 ± 1.4% (75° group), and 89.2 ± 2.3% (150° group). There was no group difference (one-way ANOVA, *F*(2,33) = 2.42, *p* = 0.104). Out of those trials, mean percentages of trials where the initial saccades landed in the target area were 92.0 ± 1.8 (SE) % (30° group), 73.9 ± 7.8% (75° group), and 86.3 ± 3.8% (150° group), indicating that the initial saccade was used to register the target location. There was no group difference (one-way ANOVA, *F*(2,33) = 2.71, *p* = 0.081).

Subsequently, the participants typically either maintained their gaze at the target area or changed it to other locations prior to the onset of reaching, and thence, the gaze fixation was maintained until the end of reaching. To determine approximately when gazes were fixated in association with the execution of hand movements, gaze locations were analyzed at 6 different timings relative to reaching: -200, -100, 0, and 100 ms relative to the onset of reaching, peak velocity, and the offset of reaching. The results of gaze locations were very similar across all timings except 200 ms prior to the onset of reaching, indicating that gazes were fixated by 100 ms before the onset of hand movement in all three groups and maintained until the hand-movement offset. Since the observed patterns of gaze locations became very stable at the onset of hand movements, we will report the gaze patterns at that timing in the rest of the Results section.

To illustrate the changes of gaze patterns due to practice, angular histograms of gaze direction errors based on all trials from all participants are shown in Fig 3g–3l for the early and late practice phases. In early practice, the peak distribution was located at ~30° for the 30° group (Fig 3g) and at ~75° for the 75° groups (Fig 3h), indicating that gazes were mainly directed at the target. However, other gaze directions of the 75° group were widely distributed across the workspace. The 150° group showed a profile with two peaks, one around the target direction (150°, Fig 3i) and the other around the 180° inverted-target direction (-30°). In late practice, the distribution of gaze directions was shifted toward 0° slightly for the 30° (Fig 3j) and conspicuously for the 75° group (Fig 3k), indicating that the participants more frequently gazed in the direction of the hand target (i.e., the hand location that brings the cursor on the target under the visuomotor rotation). The 150° group still showed the two-peak profile, but the gazing directions was shifted more toward the inverted-target direction (Fig 3l).

**Fig 5:**
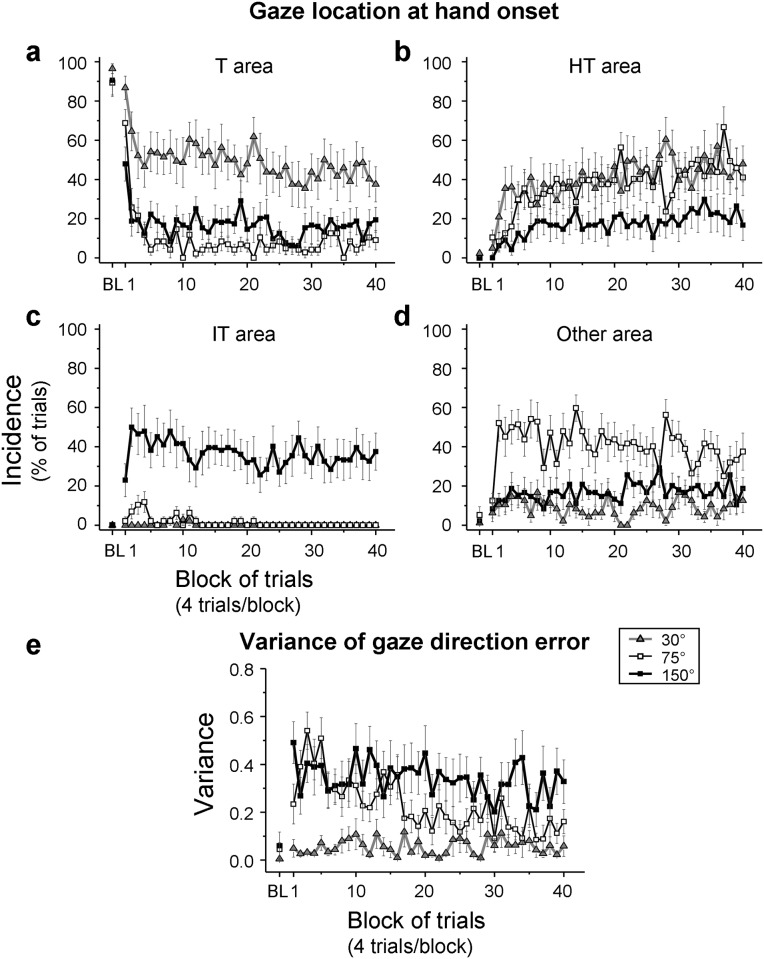
Adaptive changes of gaze behaviors during practice of a visuomotor rotation. In (a-d), mean incidences of gaze locations at the onset of reaching are plotted for 4 different areas of workspace (see Fig 2 for details): the target (T) area (a), the hand-target (HT) area (b), the inverted-target (IT) area (c), and the other area (d). The incidences were calculated across 4 trials per trial block, and mean values of all participants are plotted against 40 trial blocks for the 30° (triangles), 75° (open squares), and 150° (filled squares) groups. Mean values of baseline performance (BL) are also plotted. The error bars represent the SE. In (e), mean inter-trial variability (i.e., circular variance) of gaze direction errors is plotted in the same format. Circular variance has an arbitrary unit with 1 being the maximum and 0 the minimum.

To quantify the adaptive changes of gaze patterns throughout practice, the incidences of gazes falling in different gaze areas (Fig 2) were analyzed for each trial block of practice (Fig 5). All groups mostly looked at the target (T) area for the baseline (Fig 5a), and there was no group difference (one-way ANOVA, *F*(2,33) = 0.44, *p* = 0.646). In the first several blocks of practice, they rapidly reduced the respective incidences down to ~50% (30° group), ~10%, (75° group), and ~20% (150° group). The 30° group gazed more at the T area than did the other groups (3 (group) x 2 (phase: early vs late practice) ANOVA, group effect: (*F*(2,33) = 10.10, *p* < 0.001; post-hoc, *p* = 0.001 for both comparisons), which did not differ from each other (*p* = 0.618). The phase effect was significant (*F*(1,33) = 28.60, *p* < 0.001), while the group-by-phase interaction was not (*p* = 0.095).

Accompanying the reduced gaze incidences at the T area, all groups gradually increased the incidences of looking at the hand-target (HT) area throughout practice (Fig 5b), resulting in a significant phase effect (*F*(1,33) = 23.34, *p* < 0.001). However, there was no group effect (*p* = 0.051) or group-by-phase interaction effect (*p* = 0.399). Furthermore, the 150° group showed substantially higher incidences of gazing at the inverted-target (IT) area than did the other groups (Fig 5c, group effect: *F*(2,33) = 29.05, *p* < 0.001; post-hoc, *p* < 0.001 for both comparisons). The 75° group, in contrast, had significantly higher gaze incidences at the other areas than did the 30° and 150° groups (Fig 5d, group effect: *F*(2,33) = 8.67, *p* = 0.001; post-hoc, *p* = 0.001 for both comparisons). This was due to that the 75° group gazed at a relatively wide area across the working space in early practice (Fig 3h) and at surrounding areas of the HT area in late practice (Fig 3k). There was no phase effect and group-by-phase interaction effect for the inverted-target area (*p* = 0.868 and *p* = 0.908, respectively) and for the other area (*p* = 0.299 and *p* = 0.942, respectively). Additionally, the gaze incidences at the starting-position (SP) area throughout all practice blocks were generally low for all groups (30°: 1.6 ± 0.8% (mean ± SE); 75°: 11.3 ± 4.6%; 150°: 12.3 ± 4.5%), and there were no group, phase, and group-by-phase interaction effects (*p* = 0.156, *p* = 0.863, and *p* = 0.356, respectively).

Furthermore, to quantify progressive changes in gaze incidences for the T, HT, IT, and other gaze areas in each group, groups’ mean values were subjected to a linear regression analysis across the 40 trial blocks of practice. The 30° group (Fig 5a–5d, triangles) significantly increased the gaze incidences for the HT area during practice (*r* = 0.69, *p* < 0.001, slope = 0.59) and significantly decreased those for the T area (*r* = 0.65, *p* < 0.001, slope = -0.52). However, there were no significant trends for the IT (*r* = 0.26, *p* = 0.100, slope = -0.01) and other areas (*r* = 0.04, *p* = 0.827, slope = -0.01). The 75° group (Fig 5a–5d, open squares) significantly increased the gaze incidences across the blocks for the HT area (*r* = 0.73, *p* < 0.001, slope = 0.74) but significantly decreased those for the T (*r* = 0.35, *p* = 0.025, slope = -0.33), IT (*r* = 0.60, *p* < 0.001, slope = -0.15) and other areas (*r* = 0.32, *p* = 0.043, slope = -0.26). The 150° group (Fig 5a–5d, filled squares) significantly increased the gaze incidences both for the HT (*r* = 0.68, *p* < 0.001, slope = 0.35) and other areas (*r* = 0.38, *p* = 0.015, slope = 0.15) but significantly decreased those for the T (*r* = 0.33, *p* = 0.036, slope = -0.20) and IT areas (*r* = 0.40, *p* = 0.011, slope = -0.21).

#### Variability of gaze direction errors

To examine whether variability of gaze directions decreased with practice, gaze direction error was measured in each trial (see Fig 3g–3l). Subsequently, inter-trial variability (i.e., circular variance) of gaze direction errors was calculated for each block in each participant (Fig 5e). Mean variability across participants in the baseline was small in all groups (30°: 0.005, 75°: 0.044, 150°: 0.060). A 3 (group) x 3 (phase: baseline, early practice and late practice) ANOVA showed a significant group-by-phase interaction (*F*(4,66) = 4.79, *p* = 0.002, ɛ = 0.709). The interaction stemmed from differential variability increases among groups from the baseline to early practice (post-hoc, interaction effect of a 2 (phase) x3 (group) ANOVA, *F*(2,33) = 9.29, *p* = 0.001) and from the baseline to late practice (post-hoc, interaction effect, *F*(2,33) = 8.45, *p* = 0.001). As can be seen in Fig 5e, the 150° group substantially increased the variability from the baseline to both early and late practice, and so did the 75° group from the baseline to early practice. Conversely, the 30° group hardly showed such increases. The variability changes from the early to late phase were similar among groups (post-hoc, interaction effect, *F*(2,33) = 1.41, *p* = 0.257). There was also a significant group main effect (*F*(2,33) = 15.53, *p* < 0.001). The 30° group showed significantly smaller variability than the other groups (post-hoc, 75°: *p* < 0.001, 150°: *p* = 0.004), which did not differ from each other (*p* = 0.020). The variability was significantly greater in early practice than in the baseline (phase main effect, *F*(2,66) = 17.59, *p* < 0.001, ɛ = 0.709; post-hoc, *p* < 0.001) and late practice (*p* < 0.001), which did not differ from each other (*p* = 0.221).

In summary, all groups shifted their gaze locations from the target area to the hand-target area during practice. Besides this change, the 75° and 150° groups also gazed at the wider surrounding areas of the hand-target area and the inverted-target area, respectively. All groups reduced the variability of gaze directions with practice, but the 75° and 150° groups had greater variability than did the 30° group.

### Spatial relation between gaze and hand directions

**Fig 6:**
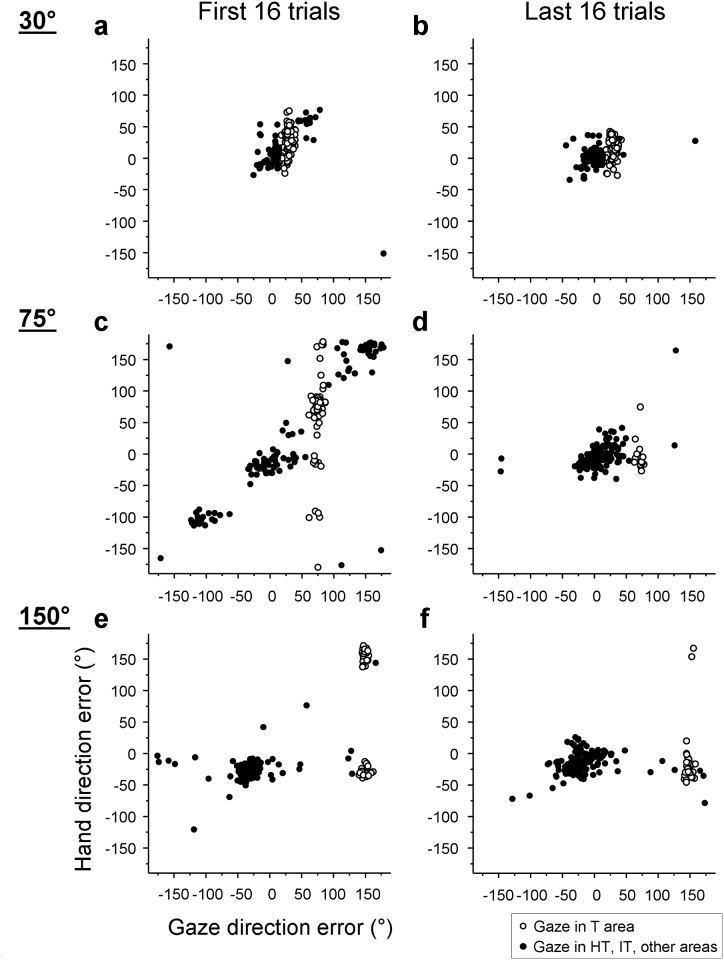
Relations between gaze and hand direction errors. Scatter plots show the relations between gaze direction errors at the onset of reaching and hand direction errors in the first 16 trials (a, c, e) and last 16 trials (b, d, f) of the practice condition. All trials across all participants except trials where gazes fell in the SP area (see Fig 2) are included in the plots. Open circles represent trials where gazes fell in the T area, whereas filled circles represent trials where gazes fell in the HT, IT and other area (see Fig 2). Plots of the 30°, 75°, and 150° groups are shown in the top, center and bottom rows, respectively.

Since gaze shifts occurred earlier than hand movements, gaze directions may reflect a predictive gaze control for hand movements [46,47]. If so, gaze directions would be related to hand directions. To explore this possibility, the relation between gaze direction errors and hand direction errors was analyzed. Note that trials where the gazes fell in the SP area as reported above were excluded from the analyses. Scatter plots of hand and gaze direction errors for the first 16 trials of practice (Fig 6a, 6c and 6e) revealed two patterns of such relation when the participants were exploring the unfamiliar visuomotor transformation. One was that the gazes were directed toward the directions of hand movements (Fig 6a, 6c and 6e), resulting in a positive correlation across trials. The other was that gazes were directed to the target area, while hands explored different directions (Fig 6a, 6c and 6e, open circles). In the last 16 trials, these patterns were less clear (Fig 6b, 6d and 6f), because hand direction errors were much more concentrated to a narrow range than in the first 16 trials.

**Fig 7:**
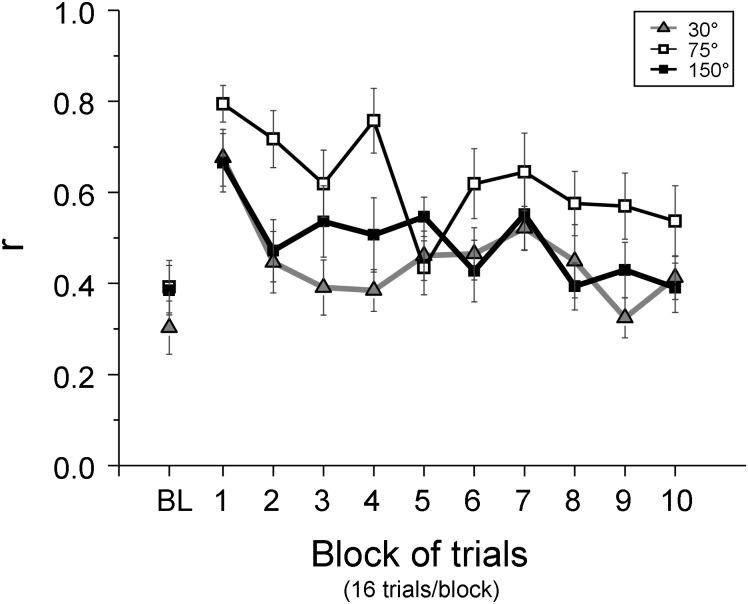
Adaptive changes of correlations between gaze and hand direction errors during practice of a visuomotor rotation. Mean values of all participants are plotted against 10 trial blocks with 16 trials each for the 30° (triangles), 75° (open squares), and 150° (filled squares) groups. Mean values of baseline performance (BL) are also plotted. Trials where gazes fell in the SP area (see Fig 2) are excluded for this calculation. The error bars represent the SE.

Next, to quantify the above relation between gaze and hand directions, we divided practice trials into 10 trial blocks of 16 trials each and computed the correlations between gaze and hand directions across trials for each participant in each block. Mean correlation coefficients in the baseline ranged between 0.3 and 0.4 across groups (Fig 7). All groups increased the correlations in early practice. However, the correlations were gradually reduced with more practice. A 3 x 3 ANOVA revealed a significant phase effect (*F*(2,66) = 27.26, *p <* 0.001) and a group effect (*F*(2,33) = 3.82, *p* = 0.032), while there was no group-by-phase interaction (*p* = 0.792). Correlations of the 75° group were significantly greater than those of the 30° group (post-hoc, *p* = 0.015), but did not differ from those of the 150° group (*p* = 0.036). The 30° and 150° groups did not differ from each other (*p* = 0.705). The correlations significantly increased from the baseline to early practice (post-hoc, *p* < 0.001), but decreased from early to late practice (*p* < 0.001). The correlations in late practice did not differ from those of the baseline (*p* = 0.085). Note that additional analysis revealed that the relatively low correlations found in the baseline and the late practice phase were likely mainly due to the hand and eye direction error variances being too small to detect correlation between them (see S1 Text and S1 Table for more details).

In summary, while the hand moved to various directions of the workspace especially in early practice, gazes were directed either toward the directions of hand movements or the direction of visual targets. The correlations between hand and gaze directions were increased in early practice, but gradually decreased with practice in all groups.

### Adaptive changes, after-effects, and explicit knowledge

#### Adaptive shift

**Fig 8:**
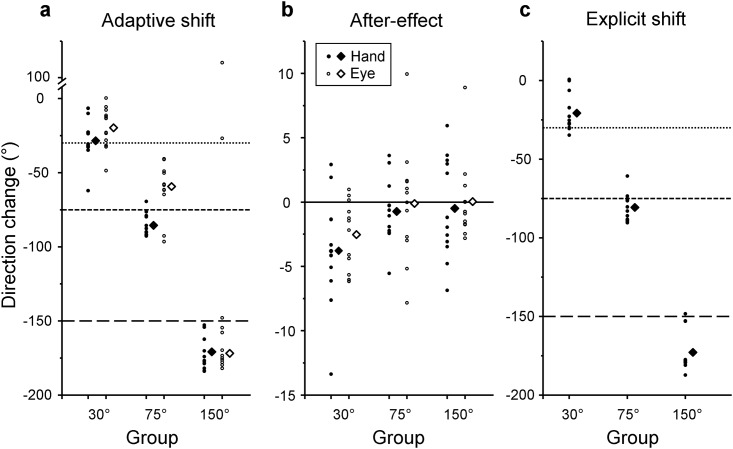
Adaptive shifts (a), after-effects (b), and explicit shifts (c). Individual values (circles) and mean values of all participants (diamonds) are plotted against groups. Filled circles and open circles refer to hand and eye movements, respectively. Horizontal dotted line, dashed line, and long-dashed line refer to the values that would be obtained when 30°, 75°, and 150° visuomotor rotations, respectively, are fully adapted.

Adaptive shift, after-effect, and explicit shift were measured to assess different types of knowledge of the visuomotor rotation acquired through practice by subtracting pre-tests from post-tests [16,26,39,40]. Since no terminal visual feedback was given during the pre- and post-tests, these measurements excluded any between-trial influence of terminal feedback. Adaptive shift measured adaptive changes of hand movements, reflecting both implicit corrections based on implicit knowledge of the visuomotor rotation and strategic corrections based on explicit knowledge of the rotation. Adaptive shifts of -30°, -75°, and -150° would completely compensate for the rotations of 30°, 75°, and 150°, respectively. Individual values and group’s mean values of adaptive shifts are shown in Fig 8a (filled circles and diamonds). The 30° group compensated the rotation fairly well (mean value: -28.4°, 1-sample t-test against -30°, *df* = 11, *p* = 0.713). In contrast, both the 75° group (mean value: -85.5°) and 150° group (mean value: -170.9°) overcompensated the respective visuomotor rotations (1-sample t-test against -75°, *t*(11) = 4.76, *p* = 0.001; t-test against -150°, *t*(11) = 6.06, *p* < 0.001). Since the participants of the 75° (or 150°) group may have adapted to the 90° axial rotation (or the 180° target inversion), a 1-sample t-test against -90° (or -180°) was also performed. The 75° group did not show significant deviation from -90° (*p* = 0.064). However, the 150° group showed significant deviation from -180° (*t*(11) = 2.67, *p* = 0.022), indicating that the overcompensation of this group was not entirely reflecting the 180° target inversion.

To examine if eye movements showed similar patterns as the hand movements, adaptive shifts of eye movements were measured based on gaze directions during the pre- and post-tests (Fig 8a, open circles and diamonds). Mean values were -19.7° for the 30° group and -59.3° for the 75° group, which were significantly smaller than the respective adaptive shifts of hand movements (Fig 8a, filled circles, 30° group: t-test, *t*(11) = 4.75, *p* = 0.001; 75° group: *t*(11) = 5.06, *p* < 0.001 for both). Conversely, the mean value for the 150° group was -171.8°, which did not differ from that of hand movements (Watson-Williams two-sample test, *p* = 0.93).

#### After-effect

After-effect measured the magnitude of implicit knowledge of the visuomotor rotation acquired through practice. After-effects of -30°, -75°, and -150° would indicate that participants acquired perfect implicit knowledge of the associated rotations. Mean after-effects of hand movements were -3.8°, -0.7°, and -0.5° for the 30°, 75°, and 150° groups (Fig 8b, filled circles and diamonds). In terms of the eye movements (Fig 8b, open circles and diamonds), mean after-effects were -2.5°, -0.1°, and 0.9° for the 30°, 75°, and 150° groups, respectively, all of which did not significantly differ from the respective after-effects of hand movements (t-test, *df* = 11, 30°: *p* = 0.408; 75°: *p* = 0.703; 150°: *p* = 0.428). Only the after-effects of the 30° group were significantly different from 0° regarding both hand (1-sampled t-test, *t*(11) = 3.05, *p* = 0.011) and eye movements (*t*(11) = 3.33, *p* = 0.007), indicating that implicit knowledge of the visuomotor rotations was acquired in both effectors.

#### Explicit shift

Explicit shift measured the magnitude of explicit knowledge of the visuomotor rotation acquired through practice. The values for -30°, -75°, and -150° would indicate that participants acquired perfect explicit knowledge of the associated rotations. All groups had substantial explicit knowledge of their respective rotations (Fig 8c). Mean value (-20.7°) of the 30° group was significantly smaller than -30° (1-sample t-test, *t*(11) = 2.64, *p* = 0.023). Conversely, the 75° group (mean value: -80.7°) and the 150° group (mean value: -172.9°) had greater magnitudes of explicit knowledge than -75° (*t*(11) = 2.23, *p* = 0.047) and -150° (*t*(11) = 5.97, *p* < 0.001), respectively. The explicit shift of the 75° (or 150°) group was also subjected to a 1-sample t-test against -90° (or -180°) to examine if the explicit knowledge is related to the 90° axial rotation (or the 180° inversion) of target direction. The 75° group had no such knowledge (*t*(11) = 3.68, *p* = 0.004), but the 150° group did (*p* = 0.083).

#### Relation between adaptive shift and explicit shift

To examine if explicit knowledge of the applied rotation was related to the magnitude of adaptation, at first the mean values of adaptive shift and explicit shift were compared in each group. In the 30° group, explicit shift (Fig 8c, filled square) was significantly smaller than adaptive shift of reaching (Fig 8a, filled square, paired t-test, *t*(11) = 2.97, *p* = 0.013), but similar to that of eye movements (Fig 8a, open square, *p* = 0.765). In the 75° group, the explicit shift was significantly smaller than the adaptive shift of reaching (*t*(11) = 2.59, *p* = 0.025), but greater than that of eye movements (*t*(11) = 4.39, *p* = 0.001). In the 150° group, explicit shifts were similar to adaptive shifts of both eye (*p* = 0.571) and hand (*p* = 0.454) movements. Second, the correlation between individual explicit shifts and individual adaptive shifts was computed in each group. The 30° group showed significant correlations both for hand (*r* = 0.77, *p* = 0.004) and eye movements (*r* = 0.69, *p* = 0.014). For the 75° and 150° groups, significant correlations were found for reaching (75°: *r* = 0.70, *p* = 0.011; 150°: *r* = 0.77, *p* = 0.004) but not for eye movements (75°: *r* = 0.43 *p* = 0.162, 150°: circular-linear correlation, *r* = 0.11, *p* = 0.935).

In summary, adaptive shifts of both hand and eye movements were mainly attributed to explicit shifts in all groups. Only the 30° group also made small aftereffects in both effectors. Adaptive shifts of eye movements were smaller than those of hand movements in the 30° and 75° groups.

## Discussion

The present study examined adaptive changes of reaching movements and gaze patterns during learning of a visuomotor rotation with three different rotation angles (30°, 75° and 150°) by using terminal visual feedback. The results showed that (1) the 30° group well adapted hand movements to the applied rotation, whereas the 75° group slightly and the 150° group substantially overcompensated the respective rotations; (2) During practice, all groups gradually shifted gaze directions from the target area to the areas related to the final positions of reaching; (3) All groups increased correlation between gaze and hand directions in early practice but gradually decreased thereafter; (4) The adaptive changes of both hand and eye movements mainly reflected explicit learning in all groups, whereas only the 30° group showed small implicit adaptation in both movements. We shall discuss these results in turn.

### Adaptive change of hand movements

The difficulty of the visuomotor rotation task was manipulated by applying different rotation angles. The 30° group steadily decreased direction errors of reaching during practice, and its variability was smallest among the groups (Fig 4), showing an ease of adapting to the smaller rotation compared to other rotations tested [16,30–33]. In contrast to the 30° group, the 75° group displayed large variability of direction errors in the first half of practice (Fig 4), indicating that the participants extensively explored the relation between the hand and cursor movements [24]. In late practice, the hand movements slightly overcompensated the applied rotation. Since adaptive shifts of this group were closer to the case of a 90° rotation rather than 75° (Fig 8), a possible explanation for the small overcompensation is that hand movements may be attracted to a perpendicular direction from the SP-Target vector, even though the participants do not have such explicit knowledge. Since horizontal and vertical contours were visible in the current study (such as edges of computer monitor, etc.), and since the visual targets were located along the diagonals, the participants were possibly reversing the visual targets around the horizontal or vertical axis to make reaching movements, resulting in a 90° shift. Such adaptive pattern was observed previously as a simplified transformation pattern in complex visuomotor transformations [37,48] (see also [49] for a visuomotor rotation).

The 150° group largely overcompensated the applied rotation, and adaptation was incomplete (Fig 4a). This was due to the involvement of a two-step rotational transformation [16,30–32]. Previous studies indicated that adaptation to rotation angles probably up to 90°-120° involved a single rotational transformation, but rotation angles near 180° (e.g., 135°, 150°) involved a two-step transformation [16,30–32]. The initial reversal shift of 180° was learned quickly in the first few trials, but adaptation to the remaining 30° was poor. In the end, the participants stabilized hand movements at a wrong reaching direction with explicit knowledge closer to 180° rotation (Figs 4 and 8). Such a poor adaptive pattern is similar to the case of learning a 1^st^ order lever transformation that also involves a two-step transformation (an initial left-right reversal shift and a subsequent amplitude transformation) under terminal feedback [48,50]. Thus, application of a strategic reversal shift of 180° seems to reduce the CNS’s capability to detect relatively small direction errors between the target and the feedback cursor for explicit adjustment [23] and between the cursor and planned hand direction for implicit adaptation [23]. Since the 30° group was able to adapt to the applied rotation, the poor adaptation to the remaining 30° rotation after the reversal-shift for the 150° group was not caused by the small rotational magnitude per se but by the application of the reversal shift. Gazes of the 150° group were often directed to the inverted-target area (Fig 5). This gaze pattern may have reduced the accuracy in registering the feedback cursor and the target locations, thereby making it difficult to detect the above direction errors.

### Adaptive change of gaze behaviors

Applying three different rotations revealed distinct differences in adaptive gaze behavior depending on the rotations. Gaze directions of the 30° group were gradually adjusted from the target area to the hand-target area during practice, and the variability of gaze directions was smallest among the groups (Fig 5), again indicating the relative ease of learning a small visuomotor rotation [16]. The 150° group shifted the gazes toward the inverted-target area in concert with a reversal shift of hand movements from very early practice, suggesting that the 180° reversal shift is easily applied without much of exploration of gaze and hand directions. In contrast, the 75° group shifted the gaze to wide areas of workspace and showed large variability of gaze directions especially in the first half of practice, reflecting the difficulty of learning this magnitude of visuomotor rotation.

One of the most informative findings of the current study was that in all groups, gaze incidences for the target area gradually decreased during practice, and in return, gaze incidences for the hand-target area increased (Fig 5). Gazes in late practice were also directed to areas surrounding the hand-target area in the 75° group or to the inverted-target area in the 150° group. These results indicate that gaze directions were altered through practice from the visual targets to the final positions of reaching. Under terminal feedback, there is no reliable means to adjust the reaching directions during movement, and hence, modifications of reaching have to be done in a pre-planned manner in subsequent trials. The observed strategy of bringing the gazes closer to the final positions of reaching under a visuomotor rotation is in a way similar to the case of reaching to a visual target in a natural environment (because the visual target and the final position of hand movement are the same). Importantly, such a gaze pattern is known to improve the accuracy of planning of hand movements [1,2,5–7,51]. Therefore, the observed adaptive gaze pattern reflects the oculomotor system’s role under terminal feedback to provide visual information most needed for the effective preplanning of reaching to counter the applied rotation.

This adaptive pattern of aligning the gaze directions on the final position of reaching is different from the one found in the continuous feedback, in which gaze behavior is gradually adjusted during practice from fixating on the feedback cursor to fixating on the visual target (where the feedback cursor should reach) [16,17]. Such gaze anchoring to the target is conducive to assess the relation between the feedback cursor and the target during reaching, guiding the cursor to the target. Thus, these different adaptive gaze-behaviors during learning of visuomotor transformations reflect two different sensorimotor processes involved in the control of reaching, namely preplanning based on terminal visual feedback and on-line processing based on continuous visual feedback.

Another informative finding of the current study was that all groups increased correlations between the reaching and gaze directions across trials to the moderate to high level when the visuomotor rotation was introduced (Fig 7). Since the gaze shifts occurred earlier than the hand movements, such correlations suggest that gazes are shifted predictively in the direction of hand movements [46,47]. In other words, the gazes lead reaches in exploring the unfamiliar visuomotor transformation. Furthermore, this gaze control pattern was supplemented by another pattern where gazes were directed to the target area while the hand moved to different directions (Fig 6). In this case, gazing at the target area likely provides a reference direction (e.g., SP-target vector), from which a voluntary shift of hand direction of a certain magnitude can be calculated to plan reaching direction [45]. After the reaching is completed, this magnitude is likely compared with direction errors between the target and the feedback cursor, so that the planning error of reaching direction can be assessed and used to adjust reach planning in the later trials. Taken together, the sensorimotor system uses two ways of controlling gaze directions relative to reaching directions in exploring unfamiliar visuomotor transformation.

Interestingly, in late practice where reaching directions had low spatial inter-trial variability (Fig 6), the correlations between gaze and hand directions across trials were reduced to a low to moderate level, as was the case in baseline performance (Fig 7). These results suggest that even though gazes point in relatively similar directions as reaches (Fig 3, LP), gaze directions cannot precisely predict trial-to-trial changes of reaching directions.

### Implicit and explicit processes involved in adaptive change

Pre- and post-tests revealed that all groups had acquired substantial explicit knowledge of the applied rotations. However, the magnitudes of explicit knowledge and that of adaptive shifts of hand movements were similar only in the 150° group (Fig 8). Nevertheless, individual explicit shifts were significantly correlated with individual adaptive shifts in all groups. These results suggest that the participants in all groups became aware of the nature of the visuomotor transformation during practice and intentionally adjusted reaching directions at the preplanning stage to compensate for the applied rotation [25,38].

Regarding eye movements, the magnitude of adaptive shifts was either similar to (for the 30° and 150° groups) or smaller than (for the 75° group) the magnitude of explicit shifts. Thus, adaptive changes of eye movements were again mainly accomplished by explicit adjustments of gaze directions based on explicit knowledge of the rotation. However, individual explicit shifts were correlated with individual adaptive shifts only in the 30° group, which differed from hand movements where adaptive shifts were correlated with explicit shifts in all groups. These results suggest that explicit adjustments of eye movements are less precise than those of hand movements to counter the applied rotation under a task requirement used in this study. Eye movements are, therefore, likely adjusted to guide the hand in the general direction that is perceived to counter the visuomotor rotation, whereas precise adjustments of reaching directions are done by the limb-motor system. Such a postulate may be supported by our previous study, which examined the role of gaze locations in learning of a visuomotor rotation where explicit adjustments of hand movements had to counter a concurrent implicit adaptation that brought the hand in an undesired direction [21]. In that case, the gazes were gradually shifted toward a direction that countered the undesired directions of hand movements so that reaching directions were adjusted to a desired direction to compensate for the applied rotation.

Only the 30° group displayed small but significant after-effects in both eye and hand movements. Thus, only the group with the smallest rotation angle created an internal model of the visuomotor rotation through practice, resulting in implicit adaptation as well [21,23,24]. The presence of after-effect in eye movements is noteworthy because it means that sensorimotor learning results in creation of an internal model of the visuomotor rotation not only for hand movements but also eye movements that support the planning and execution of manual actions.

### Factors that influence adaptive change

The presence of after-effects, and thus implicit adaptation, under the use of terminal feedback has been inconsistent in literature. After-effects were present in the 30° group and in previous studies using a 45° rotation [21,23,24]. Conversely, after-effects were absent in the 75° and 150° groups and in other previous studies using a 30° [22] and 60° rotation [18,19]. One possible factor for the discrepancy is different sizes of visuomotor rotations. Although using terminal feedback leads to smaller implicit adaptation compared to continuous feedback [24], explicit knowledge of the visuomotor rotation is generally small for small visuomotor rotations [16,35,38] and increases for larger ones [16,35,52]. Importantly, the magnitude of implicit adaptation is known to be negatively related to the awareness of the visuomotor rotation [53,54]. Thus, substantial explicit knowledge of the rotation acquired through practice due to a large rotation under the terminal feedback may have attenuated implicit adaptation, causing the discrepancy among the studies.

Another possible factor for the above discrepancy is different types of hand movement used for experiments. The current study and previous studies with no after-effects used a point-to-point movement to the target, which involved planning of the hand’s initial trajectory direction and a control for stopping at the target [18,19,22]. In contrast, the studies with after-effects used a slicing movement (i.e., moving through the target), which mainly involved planning of the hand’s initial trajectory direction [21,23,24] (see also [20] for a similar case using an out-and-back pointing movement). Different adaptive mechanisms between these two types of control have been suggested in learning a visuomotor rotation [55,56]. Furthermore, in the case of the slicing movement, the terminal feedback was displayed at the time when the hand moved beyond the distance between the starting position and the target. This means that although the task of reaching to the target was completed, the terminal feedback was displayed while the hand was still moving. That would provide participants additional information about the relation between the feedback cursor and the reaching direction, thereby helping to create an internal model of the visuomotor rotation, in turn producing a stronger after-effect.

Some of the above studies with slicing-type movements [20,21,23] also employed a paradigm developed by Mazzoni and Krakauer [20], where visual targets and the corresponding hand targets were displayed. The hand targets indicated hand locations where the cursor would reach to visual targets under a visuomotor rotation, and participants were instructed to reach to the hand targets in order to counter the applied visuomotor rotation. Displaying such hand targets is known to enhance implicit adaptation [23]. This is likely because visible hand targets would increase the precision of an error estimation between the predicted reaching direction and the visual feedback of reaching, which is thought to be essential to implicit adaptation [20,23,57]. In contrast, the current study displayed no hand targets, thereby possibly attenuating implicit adaptation.

It is worth mentioning that prism adaptation is thought to occur through realignment of a visuomotor map of the relationship between vision and proprioception related to reaching, giving rise to the aftereffects when the prisms are removed [58]. Such implicit adaptation occurs even when prisms introduce large rotations, so that participants are aware of the exposure [58,59]. It also occurs when terminal visual feedback is applied [60] or when continuous feedback is applied only in the last portion of reaching [61]. These observations seem to contradict the current results, where adaptive changes under terminal feedback were primarily through explicit learning. It should be noted, however, that there are critical differences in task performance conditions between prism adaptation studies and the current study of a visuomotor rotation. First of all, the entire visual information is shifted in prism adaptation, which is more conducive to the realignment of the visuomotor map compared to learning a visuomotor rotation where only visual feedback of hand movement is rotated and all other visual information (such as a starting point and a visual target, etc.) is veridical. Furthermore, unlike prism adaptation studies, the current study used a tool-use setting where visual targets and visual feedback were displayed on the vertical plane and hand movements were made on the horizontal plane, thereby requiring an additional coordinate transformation to link the hand and visual information [39]. Different visuomotor processes underlying reaching between natural and tool-use settings were revealed recently [49,62,63]. Moreover, the current task required controls of both the hand’s initial trajectory direction and stopping at the target, whereas prism adaptations often require mainly the control of the hand’s initial trajectory direction, such as throwing a ball [59] or rapid pointing being mechanically stopped as the finger lands on the target [60]. Taken together, the sensorimotor processing required for learning the current task was more complex than in the case of prism adaptations, thereby making it difficult to create an internal model of the applied rotation, in turn attenuating implicit adaptation.

### Type of visual feedback and adaptive change of gaze behavior

In the case of continuous feedback examined in our previous study [16], adaptive gaze behavior during learning of a visuomotor rotation was consistent among different control processes used to adapt reaching movements (i.e., implicit adaptation, on-line based control, or explicit adjustment). In the current study with terminal feedback, the adaptive changes of both hand and eye movements were derived mostly from explicit adjustments in all groups and only to a small extent from implicit adaptation in the 30° group. Consequently, the small implicit adaptation makes it difficult to identify how explicit adjustments and implicit adaptation of reaching differentially influence adaptive changes of gaze directions. Therefore, it is important to clarify this issue with experimental procedures where relatively large implicit adaptation of reaching can be observed together with explicit adjustments [24] and where little explicit adjustment of reaching is involved due to a very small visuomotor rotation (e.g., [64]) or a rotation applied incrementally (e.g., [25]). One possibility is that the magnitude of adaptive gaze shifts toward the final positions of reaching only relates to explicit adjustments. Such postulate is conceivable because our previous study with terminal feedback showed that the occurrence of explicit adjustments of reaching was related to an adaptive change of gaze directions, whereas the occurrence of implicit adaptation of reaching did not depend on prescribed gaze locations between the visual target and the final positions of reaching [21]. Another possibility is that the magnitude of gaze shifts reflects the sum of the respective magnitudes of explicit adjustments and implicit adaptation of reaching, thereby being involved in both implicit and explicit processes. Results of such experiments would clarify if and how the oculomotor system plays differential roles between implicit and explicit adaptive processes for planning of manual actions under terminal visual feedback.

A related open question is whether continuous feedback results in the presence of an after-effect of eye movements when a visuomotor rotation is removed. This question is critical for the understanding of conditions under which realignment of a visuomotor map occurs during learning of a visuomotor rotation. With terminal feedback, the after-effect of eye movements was observed together with that of hand movements in the 30° group (Fig 8b). This implies that, even though an internal model of the visuomotor rotation was created through practice, the implicit adaptation of reaching occurred in a manner that did not involve the realignment. To explain this phenomenon, one may need to consider the occurrence of implicit adaptation of another component besides the creation of an internal model of the visuomotor rotation. Such component is movement controllers that execute control laws for the hand and the eyes to bring these effectors to a desired location (that counters the rotation) predicted by that internal model. Once the controllers are adapted, both the hand and the eyes would move to that location when the rotation is removed, thereby resulting in after-effects of both eye and hand movements.

In the case of continuous feedback, it is unknown whether an after-effect similar to that in the current study occurs in eye movements. Our previous study with continuous feedback showed that gazes hardly moved towards the direction of the hand target during reaching throughout practice; instead, gazes were consistently directed to the visual target in late practice [16]. Therefore, it is possible that after-effect of eye movements is absent, whereas that of hand movements is present. Such pattern would imply that an internal model of the visuomotor rotation is created and that the implicit adaptation of reaching occurs in a manner that involves realignment of a visuomotor map. With continuous visual feedback, the CNS can obtain much more information about the relationship between vision and proprioception related to reaching than in the case of terminal feedback. Thus, the CNS might start modifying an internal model of that relationship and the corresponding movement controller for the hand, thereby implementing the realignment. Exploring this aspect is an exciting direction for future studies.

In summary, the results of this study support two conclusions: (1) that gaze directions for reaching movements change from the visual targets to the final positions of reaching during learning of a visuomotor rotation under terminal feedback, and (2) that adaptive changes of both eye and hand movements are dominated by explicit adjustments of movement directions to counter the applied rotation. The adaptive change of the gaze pattern reflects the oculomotor system’s involvement in the control of manual action, in particular, preplanning of reaching directions under terminal feedback.

## Supporting Information

S1 Table Mean values (SE) across participants for parameters related to hand and eye direction errors. (PDF)

S1 Text Influence of noise on correlation between eye and hand direction errors in the baseline and late practice. (PDF)
