# Supplementary material for: Eye-Hand Coordination during Visuomotor Adaptation with Different Rotation Angles: Effects of Terminal Visual Feedback
Source: PLoS One. 2016 Nov 3;11(11):e0164602. doi: 10.1371/journal.pone.0164602 (PMC5094587; doi:10.1371/journal.pone.0164602)
Supplement: S1 Table — (PDF) [file pone.0164602.s001.pdf]

S1 Table. Mean values (SE) across participants for parameters related to hand and eye direction errors.

	Baseline	Early practice phase	Late practice phase
30° group			
Vh Hand direction error variance	0.009 (0.001)	0.066 (0.017)	0.015 (0.004)
r^2 R-square statistics	0.129 (0.039)	0.500 (0.076)	0.196 (0.043)
$(1-r^2)$ * Vh Unexplained part of hand variance	0.007 (0.001)	0.022 (0.003)	0.012 (0.004)
75° group			
Vh Hand direction error variance	0.006 (0.001)	0.691 (0.072)	0.037 (0.013)
r^2 R-square statistics	0.191 (0.045)	0.650 (0.059)	0.355 (0.090)
$(1-r^2)$ * Vh Unexplained part of hand variance	0.005 (0.001)	0.252 (0.056)	0.014 (0.003)
150° group			
Vh Hand direction error variance	0.009 (0.001)	0.272 (0.057)	0.048 (0.020)
r^2 R-square statistics	0.181 (0.046)	0.488 (0.086)	0.185 (0.047)
$(1-r^2)$ * Vh Unexplained part of hand variance	0.007 (0.001)	0.124 (0.031)	0.036 (0.016)

Vh: circular variance of hand direction error.
r^2: the square of the correlation coefficient between eye and hand direction errors.
$(1-r^2)$ * Vh: part of Vh that cannot be explained by eye direction error due to eye-hand direction error correlation.
Baseline: last 16 trials of the baseline 2 condition.
Early practice phase and late practice phase: first and last 16 trials of the practice condition, respectively.
(Note that mean Vh values shown in this Table are slightly different from the ones reported in the main article, where those values (Fig 4b) were measured based on the last 8 trials of each task performance phase.)
