# Supplementary material for: Eye-Hand Coordination during Visuomotor Adaptation with Different Rotation Angles: Effects of Terminal Visual Feedback
Source: PLoS One. 2016 Nov 3;11(11):e0164602. doi: 10.1371/journal.pone.0164602 (PMC5094587; doi:10.1371/journal.pone.0164602)
Supplement: S1 Text — (PDF) [file pone.0164602.s002.pdf]

Influence of noise on correlation between eye and hand direction errors in the baseline and late practice

Low-to-moderate correlations between eye and hand direction errors were found in the baseline and the late practice phase in all groups (Fig. 7, BL and 10th block of practice). In this study, eye and hand directions were measured relative to the given target as direction errors. The magnitude of those errors was small in the baseline. Thus, there is a possibility that the variability of the direction errors in the baseline was dominated by noise of hand and eye movements, which resulted in low-to-moderate correlations. In other words, it is possible that the variances of direction errors (especially that for the hand) were too small to detect significant correlation between them. Thus, we have performed additional analysis of direction error variances to investigate whether the low-to-moderate correlations in the baseline were caused by noise or not.

The essence of the additional analysis was to determine a part of variance of hand direction error that can be explained by eye directional error due to correlation between eye and hand direction errors, and then focus on the remaining unexplained part of hand direction error variance. The unexplained part is due to noise and perhaps some other unobserved factors. Therefore, it can be viewed as the upper limit of noise magnitude. This calculation was performed separately for the baseline, early practice phase, and late practice phase. The direction error variance in the baseline was statistically compared to the hand direction error variance in early practice, based on the following rationale. If the baseline variance is found to be significantly greater than the unexplained part of the early practice variance, it would mean that the magnitude of hand direction errors in the baseline is significantly larger than the noise magnitude. Otherwise, the hand direction error variance in the baseline is likely too small to

detect significant correlation between hand and eye direction errors. In addition, the same comparisons were performed between the baseline and late practice phase and between the early and late practice phases to assess whether the hand direction errors during late practice were also dominated by noise.

For this analysis, we measured the following three parameters for each group of participants and each task performance period: the baseline, early practice phase, and late practice phase. First, circular variance of hand (Vh) direction errors across 16 trials was measured. The square of the correlation coefficient (r^2) between eye and hand direction errors was calculated. Next, we calculated the unexplained part of hand direction error variance as $(1-r^2)*Vh$.

Mean values across all participants for these parameters are shown in Table S1. The hand direction error variance in the baseline was found to be significantly smaller than the unexplained part of hand direction error variance in the early practice phase for all groups (paired t-test, 30°: $t(11) = 3.55$, $p = 0.004$; 75°: $t(11) = 4.42$, $p = 0.001$; 150°: $t(11) = 3.64$, $p = 0.004$). The hand direction error variance in the baseline was either similar to (paired t-test, 30° group: $p = 0.370$; 150° group: $p = 0.120$) or smaller than (75° group: $t(11) = 3.05$, $p = 0.011$) the unexplained part of the hand direction variance in the late practice phase. This result means that during the baseline, the hand direction errors were not large enough for us to detect correlation between eye and hand direction errors, thereby supporting an assumption that eye-hand direction error correlation insignificance was due to noise.

To examine if the noise factor was similarly affecting the error correlation in the late practice phase, the variances were compared between the early and late practice phases. For all groups, the hand direction error variance in the late practice phase was either similar to (paired t-

test, 30° group: p = 0.216; 150° group: p = 0.074) or smaller than (75° group: $t(11)$ = 3.57, p = 0.004) the unexplained part of the hand direction variance in the early practice phase. This result suggests that insignificant eye-hand direction error correlation in the late practice phase was likely also mainly due to noise, as in the baseline.
